# Antimicrobial Dendrimeric Peptides: Structure, Activity and New Therapeutic Applications

**DOI:** 10.3390/ijms18030542

**Published:** 2017-03-03

**Authors:** Mariano A. Scorciapino, Ilaria Serra, Giorgia Manzo, Andrea C. Rinaldi

**Affiliations:** 1 Department of Biomedical Sciences, University of Cagliari, Monserrato I-09042, CA, Italy; scorciapino@unica.it; 2 Department of Chemical and Geological Sciences, University of Cagliari, Monserrato I-09042, CA, Italy; ilaria.serra18@gmail.com; 3 Institute of Pharmaceutical Science, King’s College London, London SE1 9NH, UK; giorgia.manzo@tiscali.it

**Keywords:** antimicrobial peptides, dendrimers, peptidomimetics, biofilms, antiviral, antifungal, antitumor, model membranes, biophysical methods, NMR, molecular dynamics

## Abstract

Microbial resistance to conventional antibiotics is one of the most outstanding medical and scientific challenges of our times. Despite the recognised need for new anti-infective agents, however, very few new drugs have been brought to the market and to the clinic in the last three decades. This review highlights the properties of a new class of antibiotics, namely dendrimeric peptides. These intriguing novel compounds, generally made of multiple peptidic sequences linked to an inner branched core, display an array of antibacterial, antiviral and antifungal activities, usually coupled to low haemolytic activity. In addition, several peptides synthesized in oligobranched form proved to be promising tools for the selective treatment of cancer cells.

## 1. The Rising Tide of Antimicrobial Resistance

Antimicrobial resistance is an alarming threat to public health at the global level. Current estimates of the associated burden vary greatly depending on the method of data collation and analysis [1], but a recent report commissioned by the United Kingdom government estimated that, by 2050, 10 million people will die every year due to antimicrobial resistance unless appropriate countermeasures are taken [2]. A range of infectious microbial pathogens are already resistant to most classes of clinically usable antibiotics, strongly reducing the choices of alternative treatments, that sometimes are simply non-existent [3,4]. Increasing rates of multi-drug resistance in bacterial strains that cause common infections associated with healthcare settings and/or community-acquired have been reported worldwide. Furthermore, an array of important viral, fungal and protozoan pathogens are also progressively more resistant to currently available therapies. For instance, artemisinin resistance in malaria is spreading, and increasing levels of transmitted anti-human immunodeficiency virus (HIV) drug resistance have been detected among patients starting antiretroviral treatment [5]. “A post-antibiotic era—in which common infections and minor injuries can kill—far from being an apocalyptic fantasy, is instead a very real possibility for the 21st century”, commented a recent World Health Organization (WHO) report on antimicrobial resistance [5]. Besides the health burden that this situation poses, the societal and economic impact of antimicrobial resistance is wide and of worrying proportions. Antimicrobial resistance is a composite phenomenon due to a host of circumstances, but its rise has certainly been hastened by the selective pressure exerted by the use and misuse of antimicrobial agents in both animals and humans. The natural environment is also a vast source of antibiotic resistance, since it “harbors a diverse reservoir of resistance determinants, including resistance genes and the mobile genetic elements that operate as vectors for them” [6]. In addition to the need to preserve the effectiveness of existing antibiotics through a wiser use of their properties, the scarcity of new antimicrobial agents on the horizon to replace those that have become ineffective calls for the urgent quest for new anti-infective substances to be developed and deployed in the clinic.

## 2. Dendrons to the Rescue!

Clearly, no magic bullets exist here. Rather, several strategies aimed at replenishing our waning arsenal of anti-infective agents must be pursued. A group of substances that has drawn considerable and growing attention in the last few decades is made of antimicrobial peptides (AMPs), a vast array of molecules active to various degrees against bacteria, enveloped viruses, protozoa and fungi. Synthesized on ribosomes and gene-encoded, AMPs are a key component of the innate immune system of all metazoans and are usually cationic, fold into amphipathic structures and destabilize and/or permeate the plasma membranes of target cells [7,8,9,10,11]. Dedicated web-based repositories, such as the Antimicrobial Peptide Database [12], strive to keep up the pace of discovery of new peptides from many natural sources.

Although AMPs have good potential to be a viable alternative to conventional antibiotics, thanks for example to their tendency to rarely elicit antimicrobial resistance, they are not devoid of drawbacks. Indeed, because of their peptidic nature, they are characterized by scarce bioavailability and suffer from poor proteolytic stability, two features that have severely hampered clinical progress to date. In the attempt to overcome these disadvantages, various modifications have been introduced to develop synthetic analogues that mimic the properties of AMPs [13,14]. AMPs found in nature remain the principal model for these modifications, which are generally focused on their main physico-chemical and membrane activity-related properties, such as positive charge and amphiphilic nature.

**Figure 1:**
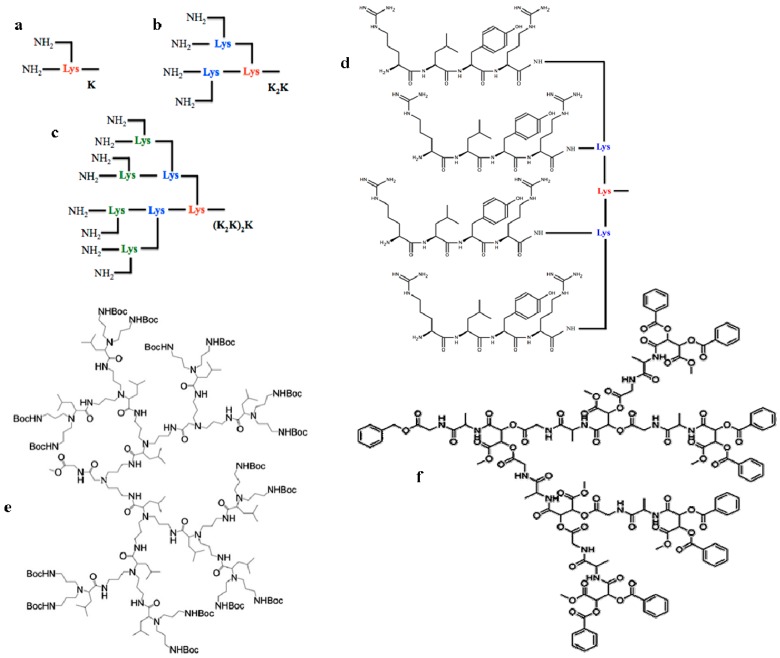
Schematic representations of three types of dendrimeric cores with three generations of lysines shown in different font styles: (**a**) Two-branched Lys (red); (**b**) Four-branched (Lys)2Lys (red-cyan); (**c**) Eight-branched [(Lys)2Lys]2Lys (red-cyan-green); (**d**) (Lys)2Lys core with α and ε branches bearing peptides Arg-Leu-Tyr-Arg; (**e**) An example of peptide dendrimer with l-Leucine dendrimer as the branching unit; (**f**) A depsipeptide dendrimer, a chiral dendrimer owing its name to its structural resemblance to depsipeptides. Arg: arginine; Leu: leucine; Tyr: tyrosine; Lys: lysine. From [17,18].

Among the most promising synthetic or semi-synthetic approaches to mimic the properties of AMPs, multimeric peptides have proven to be an intriguing variation. As reported more extensively below, several examples of peptidic dendrimers endowed with antimicrobial properties were recently reported. These molecules were in general designed following the so-called multiple antigenic peptide (MAP) system, first evolved in the 1980s as immunogens for producing site-specific polyclonal and monoclonal antibodies [15,16], where multiple peptide sequences can be added using standard solid phase chemistry to an inner core that consists of a divalent lysine (Lys) whose α- and ε-amines double geometrically with each branching generation (Figure 1). Other types of peptide dendrimers are functionalized at the periphery with peptide chains, or grafted with either unnatural amino acids or organic groups as the branching core and peptides or proteins attached as surface functional groups [17]. Figure 1 shows examples of dendrimeric peptides built around Lys residues at their core, with l-Leucine as branching units, or developed to structurally resemble depsipeptides. Overall, dendrimeric peptides show increased activity compared to monomeric counterparts, which is probably attributable to their multivalent nature, i.e., a higher local concentration (effective molarity) of bioactive units for dendrimeric molecules, as well as reduced susceptibility to the action of proteases and peptidases, a fact attributed to the nature of the branching core that with its steric hindrance would limit the cleavage rates of plasma peptidases, thus improving the peptides’ pharmacokinetics properties. The multivalency of peptide dendrimers appears to be functional to the design of AMPs for their ability to amplify cationic charges and hydrophobic clusters as the number of dendrimer branches increases, mimicking the mechanisms of action through which high-ordered antimicrobial peptides exert their membranolytic effects [18]. Since the antimicrobial potency of AMPs is by no means related to their length, with several examples of very active short peptides being known [19], the selection of short sequences for the design of antimicrobial dendrimers is desirable. Besides involving smaller production costs and less cumbersome synthetic routes, peptide dendrimers with low molecular mass have the additional advantage of being less immunogenic than high-molecular-mass dendrimers. Thus, short dendrimeric peptides may well be a useful and peculiar biopolymer design for effecting an array of membranolytic activities.

## 3. Dendrimeric Peptides as New Antibacterial Drugs

Most studies on the development of dendrimeric peptides as therapeutic agents, so far, have focused on bacterial targets. This is understandable, of course, given the clinic relevance of this type of infection. Of particular interest is the activity of several new compounds against multi-drug resistant (MDR) bacteria and biofilms. Concern regarding biofilm-associated infections is swiftly increasing worldwide, indeed, as biofilms are inherently tolerant and resistant to most antimicrobial agents. Furthermore, they have a tendency to grow on the surfaces of medical devices, considerably expanding the risk of microbial dissemination within the host and the further spread of hospital-acquired infections [20,21]. The need to deploy additional bactericidal measures is particularly urgent for biofilms, and many AMPs, both of a dendrimeric nature or not, have been tested for their ability to target specific features of sessile bacterial communities [22]. A dedicated database, the Biofilm-active AMPs database (BaAMPs) [23], is available, which reports information on the antibiofilm activity of AMPs in an organized framework.

About a decade ago, in one of the first steps along this research avenue, Neville Kallenbach and colleagues have synthesized a group of dendrimeric peptides containing multiple R (Arg) W (Trp) dipeptides [24]. The dendrimer with the best activity, named (RW)4D, was found to preferentially kill Gram-negative bacteria relative to Gram-positive strains, probably through a membranolytic mechanism. As noted by the researchers, both arginine (Arg) and tryptophan (Trp) residues occur frequently in AMPs, often in the same sequence (e.g., indolicidin), with Arg providing cationic charges that are crucial for peptide binding to the negatively-charged cell walls and membranes of bacterial targets and the lipophilic Trp anchoring peptides to the outer leaflet of the membrane and perturbing its integrity. More recently, the activity of the novel antimicrobial peptide dendrimer G3KL, with natural lysine and leucine residues alternating in the branches, against a number of *Acinetobacter baumannii* and *Pseudomonas aeruginosa* strains was reported as compared to the activities of standard antibiotics [25]. Overall, G3KL displayed a promising antibacterial activity against a large collection of difficult-to-treat isolates, including several producing carbapenemases, that are frequently faced in the contemporary international clinical scenario; in addition, the compound had little toxicity for red blood cells in vitro [25]. G3KL, which is believed to act as a membrane-disrupting compound, is a peptide dendrimer of third generation with the sequence (KL)_8_(*K*KL)_4_(*K*KL)_2_*K*KL (*K* = branching lysine), structured as multiple short dipeptides connected by branching Lys residues, and has been derived through a process of sequence optimization of a hit compound spotted by screening a combinatorial library of dendrimers, by means of a custom-made high-throughput screening assay [26,27].

**Figure 2:**
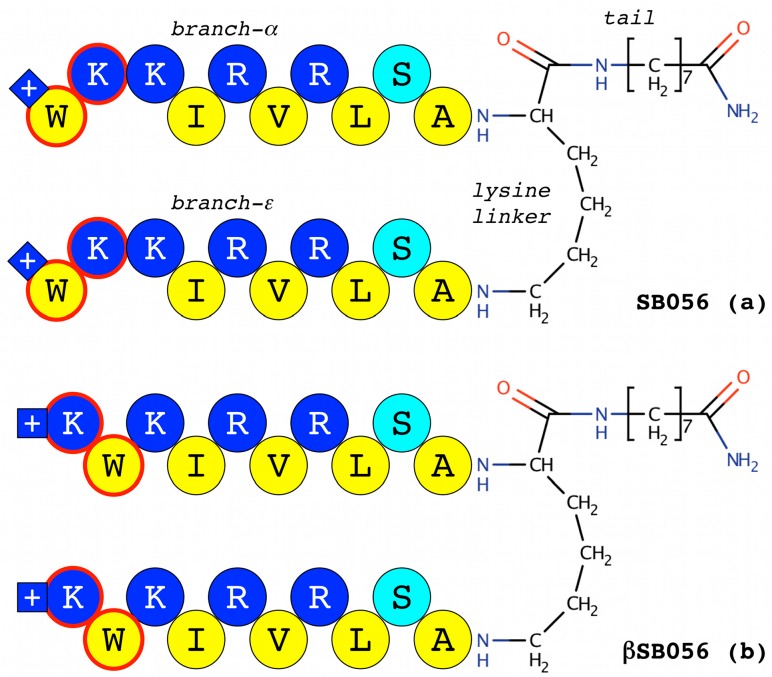
Schematic representation of the branched peptides SB056 and βSB056 (see the main text for the details). Two peptide branches are bonded to the α- and ε-amino group of a lysine residue, i.e., the linker, respectively. An 8-aminooctanamide tail is bonded to the C-terminus of the linker. The residue type is colour coded as follows: hydrophobic in yellow, positively charged in blue and polar uncharged residues in cyan. The positively-charged N-termini are represented by the blue square with the white cross in the middle. The two residues, the position of which was exchanged moving from SB056 to βSB056, are red bordered. From [30]. Reproduced with permission.

Another interesting group of antimicrobial dendrimeric peptides was derived from a short linear sequence, identified in the first place by selecting a random phage library against *Escherichia coli* cells: a subsequent rational modification and optimization process permitted obtaining the compound known as SB041, a tetra-branched peptide [28]. Further modifications of the primary sequence led to SB056, with a dimeric dendrimer scaffold. In SB056, the core is restricted to a single Lys residue, and only two copies of the same highly cationic 10-mer polypeptide are present, coupled to an octanamide tail linked to the C-terminus. This compound is highly active against Gram-negative microorganisms, with an intensity comparable to that of colistin and polymyxin B; however, it demonstrates a more extensive range of action, with some activity also against Gram-positive bacteria [29]. The use of a range of biophysical tools like circular dichroism (CD), nuclear magnetic resonance (NMR) and molecular dynamics (MD) simulations, joined with membrane affinity assays by lipid monolayer surface pressure experiments, uncovered that this intriguing peptide was indeed membrane-active by folding into a β-type conformation in lipidic environments [29]. A close inspection of the primary sequence [WKKIRVRLSA] of the peptidic part of the dendrimeric SB056 reveals a distinct pattern of alternating hydrophilic and hydrophobic amino acids, with the exception of the first two residues (Figure 2).

**Figure 3:**
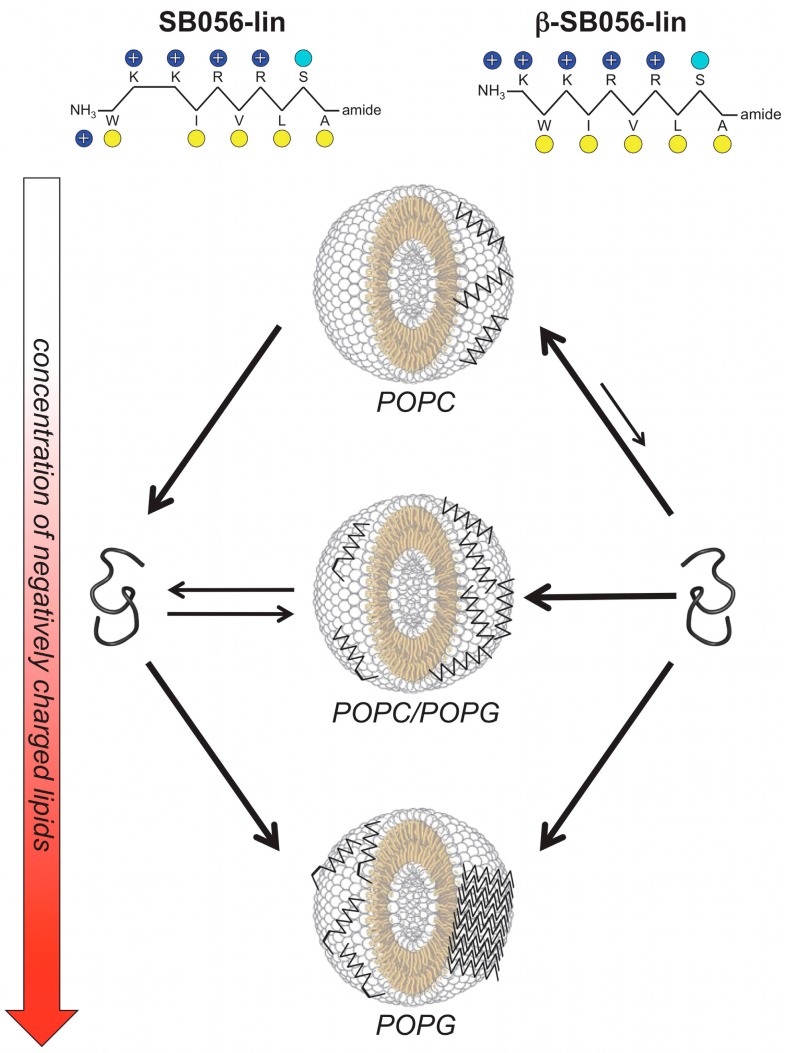
Summary of SB056 peptide-lipid interactions. The proportion of anionic lipids in the vesicles is increased from top to bottom (as indicated by the red arrow). The behaviour of the original monomeric, linear peptide SB056-lin, is represented on the left-hand side and the sequence-optimized βSB056-lin on the right. Black arrows of different lengths and thicknesses are used to indicate the different binding equilibria. SB056-lin binds only to anionic bilayers, and in a not-so-well-ordered β-stranded conformation. The sequence optimized βSB056-lin, on the other hand, binds even to zwitterionic bilayers and forms regular β-strands that self-assemble into extended β-sheets when the negative charge of the bilayer exceeds the electro-neutrality of the peptide-lipid system. POPC: 1-palmitoyl-2-oleoyl-*sn*-glycero-3-phosphocholine; POPG: 1-palmitoyl-2-oleoyl-*sn*-glycero-3-phospho-(1′-rac-glycerol). From [31]. Reproduced with permission.

**Figure 4:**
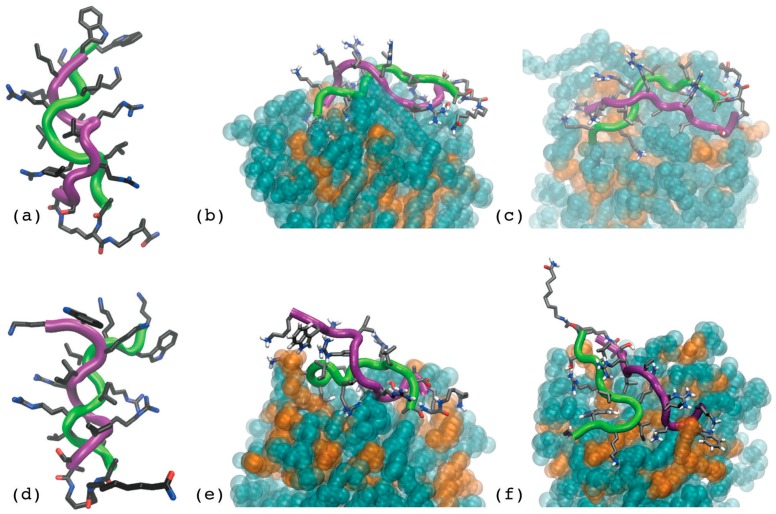
Structures obtained for SB056 (**a**–**c**) and (**d**–**f**) βSB056 in a dispersion of mixed dodecylphosphocholine (DPC)/sodium dodecyl sulphate (SDS) 3:1. These two detergents are synergistic and form mixed micelles, with the selected molar ratio being typically employed to mimic a bacterial plasma membrane [11]. First, a computer-simulated annealing was applied in a vacuum (**a**,**d**) with the distance restraints derived from experimental NOEs (cross peaks from Nuclear Overhauser Effect spectroscopy). Then, an equilibrium molecular dynamics (MD) simulation was run with the same restraints and in the presence of explicit water and detergent molecules. The last frame after 100 ns is shown in (**b**,**e**). Finally, a second 100-ns MD run was performed after releasing all restraints, and the last frame is shown in (**c**,**f**). The backbone of the α-and ε-branch is represented with a differently coloured trace, green and purple, respectively. The atoms of the detergents are represented with the corresponding van der Waals spheres. DPC and SDS are coloured in cyan and orange, respectively. From [30]. Reproduced with permission.

Given the ascertained β-type conformation of SB056, further modification of the peptidic part of the dendrimeric SB056 was then performed by interchanging the first two residues to obtain a more regular β-type conformation with a full pattern of alternating hydrophilic and hydrophobic amino acids; thus, a perfectly amphipathic analogue [KWKIRVRLSA] was designed (Figure 2). Noteworthy, such improvement of the amphipathic profile resulted in the formation of more ordered and stable β-strands when the monomeric, linear peptide interacted with model membranes and a stronger antimicrobial activity against both Gram-positive and Gram-negative bacterial strains (Figure 3) [31]. Further results obtained against *Escherichia* *coli* and *Staphylococcus aureus* planktonic strains, in the presence or without salts at physiological concentrations, corroborated the idea that the dendrimeric design is endowed with added value over the linear one, especially at physiological ionic strength, and displayed the full impact of the higher amphipathicity obtained through sequence optimization on improving peptide performances [32]. SB056 peptides also showed intriguing antibiofilm properties, especially against *Staphylococcus epidermidis* [32]. For all peptides, membrane affinity increased with the amount of negatively-charged lipids in model membranes and was less influenced by the presence of salt in the case of dendrimeric peptides. Furthermore, the amphipathically optimized analogue of SB056 displayed the highest overall affinity, even for zwitterionic 1-palmitoyl-2-oleoyl-*sn*-glycero-3-phosphocholine (POPC) bilayers, clearly indicating that, in addition to electrostatics, the distribution of hydrophobic and charged/polar residues along the primary sequence plays a significant role in driving peptide-lipid interaction [32]. Finally, a combination of NMR and MD simulations allowed the determination of the 3D structure of the two SB056 analogues, unveiling a singular β hairpin-type structure determined by the peptide chains only, with the octanamide tail eventually available for further functionalization to add new potential properties without affecting the structure (Figure 4) [30].

Another interesting example of dendrimeric peptide with antibiofilm activity is 2D-24, a new synthetic compound containing RWR (Arg-Trp-Arg) and RTtbR(2) tripeptide branches [33]. This molecule was found able to kill biofilm cells of two strains of *Pseudomonas aeruginosa*, namely the wild-type PAO1 and its mucoid mutant PDO300, in a dose-dependent manner, and also to be active against multidrug-tolerant persister cells of both strains [33].

## 4. Viruses and Fungal Pathogens as Targets

**Figure 5:**
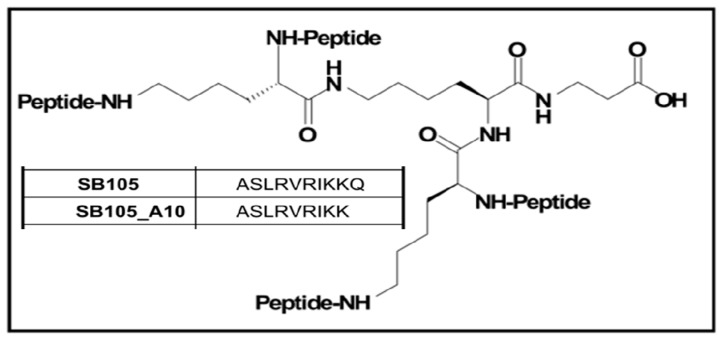
Structures of SB105 and SB105-A10 antiviral peptide-derivatized dendrimers. The generic structure of the peptide is shown. These molecules were synthesized by the addition of four 10-mer peptide chains to a tetrameric lysine central core. The amino acid sequence of each dendrimer peptide functional group is shown in the inset. See the main text for further details.

In addition to targeting bacterial pathogens, peptide-derivatized dendrimers have the capacity for other anti-infective applications, including as antiviral agents [34]. For instance, a study has recently demonstrated that the tetrabranched SB105 and its derivative SB105-A10 (Figure 5) were capable of inhibiting the replication of several strains of human cytomegalovirus (HCMV) in both primary fibroblasts and endothelial cells [35]. HCMV is a challenging pathogen for recipients of bone marrow and solid-organ transplants and among immunocompromised acquired immune deficiency syndrome (AIDS) patients: these infections are indeed a serious cause of morbidity and mortality for these groups. It seems that, in this case, dendrimers exercised their inhibitory effect by preventing the attachment of virions to heparan sulphate on the cell surface, a so far unreported antiviral mechanism that could render SB105 and SB105-A10 attractive candidates as examples of an original class of antiviral drugs [35]. The same research group reported successful use of the same dendrimeric peptides SB105 and SB105-A10 in binding heparan sulphates on cell surfaces and inhibiting the infectivity of genital types of human papillomavirus (HPV) in both 293TT and keratocarcinoma cells [36], an important finding for the potential development of a topical microbicide against sexually-transmitted viral infections. More recently, SB105 and SB105-A10 were shown to be able to directly inhibit herpes simplex virus 1 (HSV-1) and HSV-2 in vitro replication, even in conditions mimicking the physiological properties of the vagina, such as low pH values and in the presence of 10% human serum proteins. According to researchers, this new antiviral activity is based on the binding of the dendrimers to the glycosaminoglycan moiety of cell surface heparan sulphate proteoglycans (HSPGs), thereby blocking virion attachment to target cells [37]. The same mechanism seems to apply for the ability of SB105-A10 in contrasting infection of several cell lines by respiratory syncytial virus (RSV), which also interacts with HSPGs to enter target cells [38]. The potential of SB105-A10 to act as an antiviral agent was also evaluated using a model of the epithelial tissue of the human respiratory tract, made of cultured tracheal/bronchial epithelial cells; SB105-A10 markedly reduced RSV infectivity and showed no evidence of inducing cytotoxicity or proinflammatory effects, making this peptide an interesting lead for further improvement as an RSV inhibitor to be dispensed by aerosol delivery, the researchers concluded [38].

Another interesting example of a peptide dendrimer endowed with antiviral activity belongs to the group of dendrimers functionalized at the periphery with peptide chains. Marcus Weck, from the University of New York, and colleagues started from a membrane-interacting peptide from the herpes simplex virus (HSV) type 1 glycoprotein H, namely gH625–644, to complete the synthesis of a poly (amide)-based dendrimer functionalized at the termini with such a sequence [39]. The same researchers had previously conducted a thorough study of the regions of the herpes simplex virus type I glycoprotein H that have membrane-interacting capabilities, selecting the gH625–644 domain as a good candidate for the purposes of developing an antiviral agent based on its membranotropic nature: this sequence is mainly hydrophobic in nature, displaying a clear amphiphilic character when in an α-helical form, and it folds easily into a helix in a membrane-like, lipidic environment [40]. The peptidodendrimer inhibited both HSV-1 and HSV-2 at the initial phases of the entry process, probably by interacting with the glycoproteins that form the viral envelope, thus precluding the virus from closely approaching the cellular membranes, a necessary prerequisite of viral internalization [39]. For other virus-related applications of synthetic peptide dendrimers, for example as diagnostic tools and for the development of vaccines, interested readers are directed to a recently published review [41].

**Figure 6:**
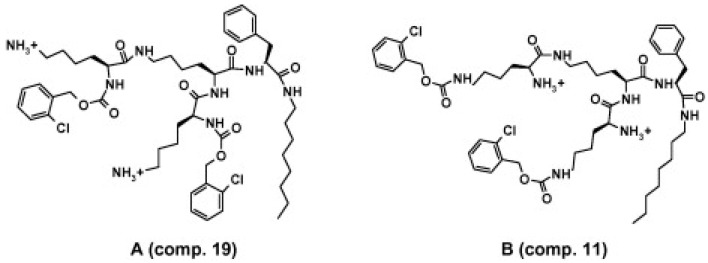
General structure of antifungal lipopeptides developed by Zofia Urbánczyk-Lipkowska and colleagues: (**A**) More branched (Compound 19); and (**B**) less branched (Compound 11). From [42]. Reproduced with permission.

**Figure 7:**
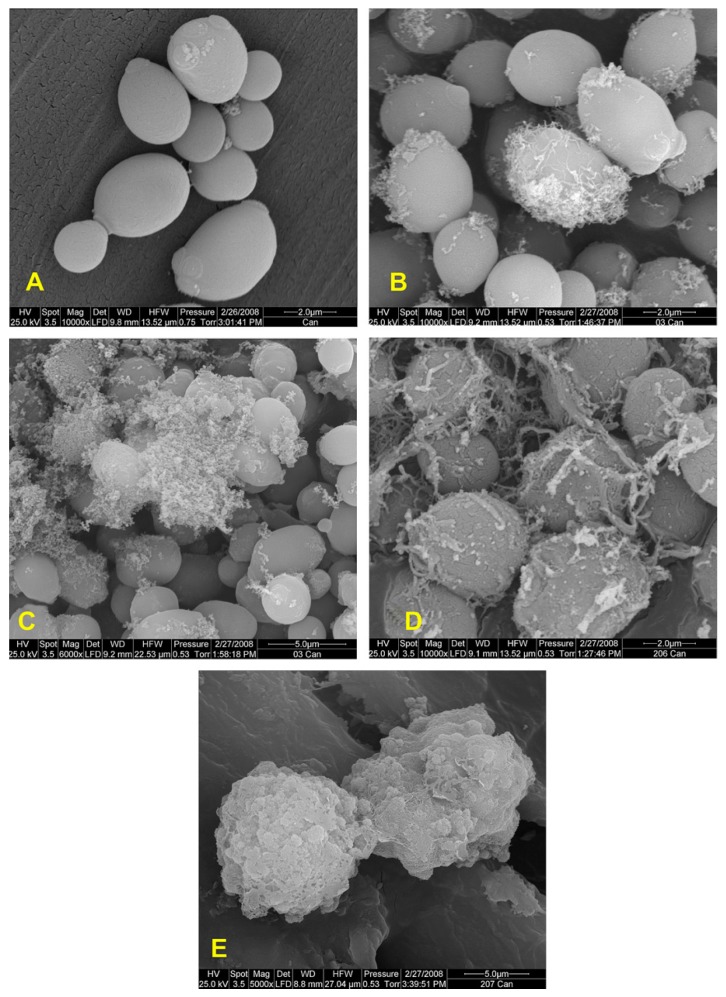
Scanning electron microscope image of *Candida albicans* cells treated with dendrimeric lipopeptides developed by Zofia Urbánczyk-Lipkowska and colleagues. (**A**) Intact cells; (**B**–**C**) Cells after exposure to lipopeptide 12 at 4 μM (0.5× minimal inhibitory concentration (MIC)) and at 10 μM (1.5× MIC); (**D**–**E**) Cells after exposure to lipopeptide 16 at 4 μM (0.5× MIC) and at 64 μM (8× MIC), respectively. From [42]. Reproduced with permission.

The group of Zofia Urbánczyk-Lipkowska at the Gdansk University of Technology, Poland, is taking a distinctive approach in designing dendrimeric peptides with potent antifungal activity. In one study, researchers synthesized a series of cationic lipopeptides of novel design, made of a branched, amphiphilic polar head derived from the (Lys)Lys(Lys) dendron and containing a C8 or C12 chain at the C-end (Figure 6) [42]. Several of the new compounds revealed an interesting activity against yeasts from the *Candida* genus; the antifungal activity of branched lipopeptides proved to be concentration dependent, and compounds caused the leakage of potassium ions from fungal cells, inducing at the same time morphological alterations (Figure 7) and inhibited activity of candidal β(1,3)-glucan synthase [42]. “Introduction of a lipid tail at C-end (particularly C12) yielded several compounds with enhanced antimicrobial potency, low hemotoxicity and selectivity shifted towards yeasts of the *Candida* genus”, the researchers commented. More recently, the same team synthesized a bunch of novel peptide dendrimers rich in Trp and with an amphiphilic fold, variable in structure and hydrophobicity, and tested their activity versus various *Candida* species and strains. A specific lead, dendrimer 14, containing four Trp residues and a dodecyl tail, was found to be particularly active, causing membrane damage at fungicidal concentration (16 μg/mL) and inducing both cell death in *Candida* cells by both apoptosis and necrosis [43]. Confocal microscopy observations showed that dendrimer 14 affects the morphogenesis and biofilm viability of *Candida*, destroying mature biofilm by acting negatively on the germination and reducing hyphae formation and also inhibited the biofilm formation [43]. Another compound with a branched structure, D186, containing four Trp residues, exhibited an anti-*Candida* action mode associated with a reduced virulence in terms of adhesive ability and pathogenic potential related to the expression of the *SAP5* gene and morphogenetic factors *EFG1* and *CPH1* [44].

## 5. Turning Branched Peptides into Antitumor Therapeutics

In dendrimeric peptides, there is more than meets the eye. A clear demonstration is the recent opening of a new research avenue, aimed at transforming protease-resistant multimeric peptides in tumour targeting agents. In particular, the group of Luisa Bracci at the University of Siena has focused on the potential applications of tetra-branched peptides (NT4) containing the human regulatory peptide neurotensin (NT) sequence. Since neurotensin receptors are overexpressed in several human tumours, like prostate, colon, pancreatic and small-cell lung cancer, researchers have explored the possibility of using NT4 conjugated to different functional units for tumour treatment and imaging.

Drugs, such as 6-mercaptopurin, combretastatin A-4 and monastrol, were conjugated to oligobranched neurotensin peptides through several linkers, and different human cancer cell lines were used to study the mode-of-release and cytotoxicity [45]. Results showed that branched peptides armed with combretastatin A-4 displayed significant activity against pancreas and prostate human cancer cells [45]. Furthermore, NT4 conjugated to methotrexate or 5-fluorodexoyuridine, which resulted in having the best in vitro cytotoxic activity, were tested in tumour xenografted mice models and were shown to reduce tumour growth by 60% and 50%, respectively [46]. Thus, “branched peptides may increase the selectivity of small molecules towards tumor cells, acting as ‘Trojan horses’ that selectively transport chemotherapy drugs into tumor cells, since they have the ability of turning nonspecific cytotoxic drugs into tumor-selective agents, which presently have a limited use in oncology due to their low selectivity and high nonspecific toxicity” [47].

More recent observations led researchers to understand that the significantly higher binding of NT4 peptides relative to the monomeric counterparts to cancer cell lines and cancer surgical samples is generated by a modification of selectivity that becomes directed toward additional membrane receptors specifically expressed by a number of different human cancers. In detail, the multimeric design provides NT4 with the ability to bind heparin and receptors belonging to the low density lipoprotein receptor (LDLR) family, which is well known to have a role in cancer biology [48]. Systematically modifying the neurotensin sequence in NT4 peptides led to the detection of a multimeric positively-charged motif mediating the interaction with both heparin and endocytic receptors [48]. A further therapeutic target for the use of branched peptides and anti-cancer agents has been bladder cancer, which ranks ninth in worldwide cancer incidence. NT4 was tested on HT-1376 and T24 bladder cancer cell lines in vitro and on human cancer samples obtained from patients that underwent radical cystectomy or endoscopic transurethral resection of the bladder, comparing results with those obtained with the healthy tissue counterpart of the same patients to evaluate its ability to recognize specific membrane receptors and to be internalized. NT4 conjugated to fluorophores distinguished cancer and healthy tissues with good statistical significance, and NT4 conjugated to methotrexate or gemcitabine was found to be cytotoxic for human bladder cancer cell lines at micromolar concentrations [49].

## 6. Dendrimers in Perspective

“Antibiotic resistance and the collapse of the antibiotic research-and-development pipeline continue to worsen despite our ongoing efforts on all these fronts. If we're to develop countermeasures that have lasting effects, new ideas that complement traditional approaches will be needed,” remarked David Gilbert and colleagues on the evolution of antibiotic resistance and strategies for combating it [50]. Making the need for action more urgent is the recent emergence of *Escherichia coli* resistance to colistin (polymyxin E), often used as the last resort for the treatment of infections caused by multidrug-resistant gram-negative bacteria; due to the *mcr-1* gene, this resistance is rapidly spreading [51,52].

As this review has strived to demonstrate, dendrimeric peptides have the potential to contribute to the development of those next-generation antibiotics we need so badly, offering virtually infinite synthetic possibilities coupled to the tendency to hit novel targets in their microbicide action and to evoke little resistance. In addition, we are probably only beginning the exploration of their versatility as clinically-applicable agents, namely in anti-cancer therapies.

As far as antibiotic resistance is concerned, needless to say, refilling the pipeline with new drugs is just one component of a global, comprehensive strategy that must be enforced in order to address the microbial resistance crisis. Other key interventions include the prevention of infections in the first place, the elimination or restriction of the use of antibiotics to promote livestock growth, the sequestration of host nutrients to prevent microbial access to nutrients, the development of other therapies focusing on host targets rather than microbial targets to avoid selective pressure driving resistance, and much more [50].
